# Ataxia in children: early recognition and clinical evaluation

**DOI:** 10.1186/s13052-016-0325-9

**Published:** 2017-01-13

**Authors:** Piero Pavone, Andrea D. Praticò, Vito Pavone, Riccardo Lubrano, Raffaele Falsaperla, Renata Rizzo, Martino Ruggieri

**Affiliations:** 1 University-Hospital “Policlinico-Vittorio Emanuele”, University of Catania, Catania, Italy; 2 Department of Clinical and Experimental Medicine, Section of Pediatrics and Child Neuropsychiatry, University of Catania, Catania, Italy; 3 Department of Biomedical and Biotechnological Sciences, University of Catania, Catania, Italy; 4 Department of Orthopaedics, University of Catania, Catania, Italy; 5 Department of Pediatrics, University “La Sapienza”, Rome, Italy; 6 Unit of Pediatrics and Pediatric Emergency, Azienda Ospedaliera Universitaria Vittorio Emanuele-Policlinico, University of Catania, Italy, Via Plebiscito 767, 95123 Catania, Italy

**Keywords:** Ataxia, Diagnostic maneuvers, Acute cerebellitis, Cerebellar syndrome, Cerebellar malformations

## Abstract

**Background:**

Ataxia is a sign of different disorders involving any level of the nervous system and consisting of impaired coordination of movement and balance. It is mainly caused by dysfunction of the complex circuitry connecting the basal ganglia, cerebellum and cerebral cortex.

A careful history, physical examination and some characteristic maneuvers are useful for the diagnosis of ataxia. Some of the causes of ataxia point toward a benign course, but some cases of ataxia can be severe and particularly frightening.

**Methods:**

Here, we describe the primary clinical ways of detecting ataxia, a sign not easily recognizable in children. We also report on the main disorders that cause ataxia in children.

**Results:**

The causal events are distinguished and reported according to the course of the disorder: acute, intermittent, chronic-non-progressive and chronic-progressive.

**Conclusions:**

Molecular research in the field of ataxia in children is rapidly expanding; on the contrary no similar results have been attained in the field of the treatment since most of the congenital forms remain fully untreatable. Rapid recognition and clinical evaluation of ataxia in children remains of great relevance for therapeutic results and prognostic counseling.

## Background

Ataxia in children is a common clinical sign of various origins consisting of impaired coordination of movement and balance with a lack of muscle control during voluntary activity [1, 2]. Ataxia is most frequently caused by dysfunction of the complex circuitry connecting the basal ganglia, cerebellum and cerebral cortex, and this type of involvement is recorded as “cerebellar ataxia.” The term “sensory ataxia” [3, 4] refers to the dysfunction of the proprioceptive sensory activity correlated with the peripheral nerves or to the posterior columns of the spinal cord.

Clinical signs in cerebellar ataxic patients are related to impaired localization. Focal dysfunction of the cerebellar vermis displays truncal unbalance, nystagmus and head waddling, and the impairment of the cerebellar hemispheres results in an anomalous gait veering toward the affected side, with asymmetry of the ipsilateral extremities and a high-stepping gait. Involvement of afferent/sensory ataxia manifests as a stepping gait and sensory damage of the extremities.

### How to recognize ataxia in children: common signs and maneuvers

Cerebellar ataxia may manifest with several signs and symptoms, the most common being a staggering gait, asynergic movements, dysmetria, nystagmus, intentional tremor and difficulty in forming speech. A through history and a careful clinical examination are important ways to recognize anomalous movements and to distinguish involvement of the cerebellum from other affected areas of the nervous system.

**Fig. 1:**
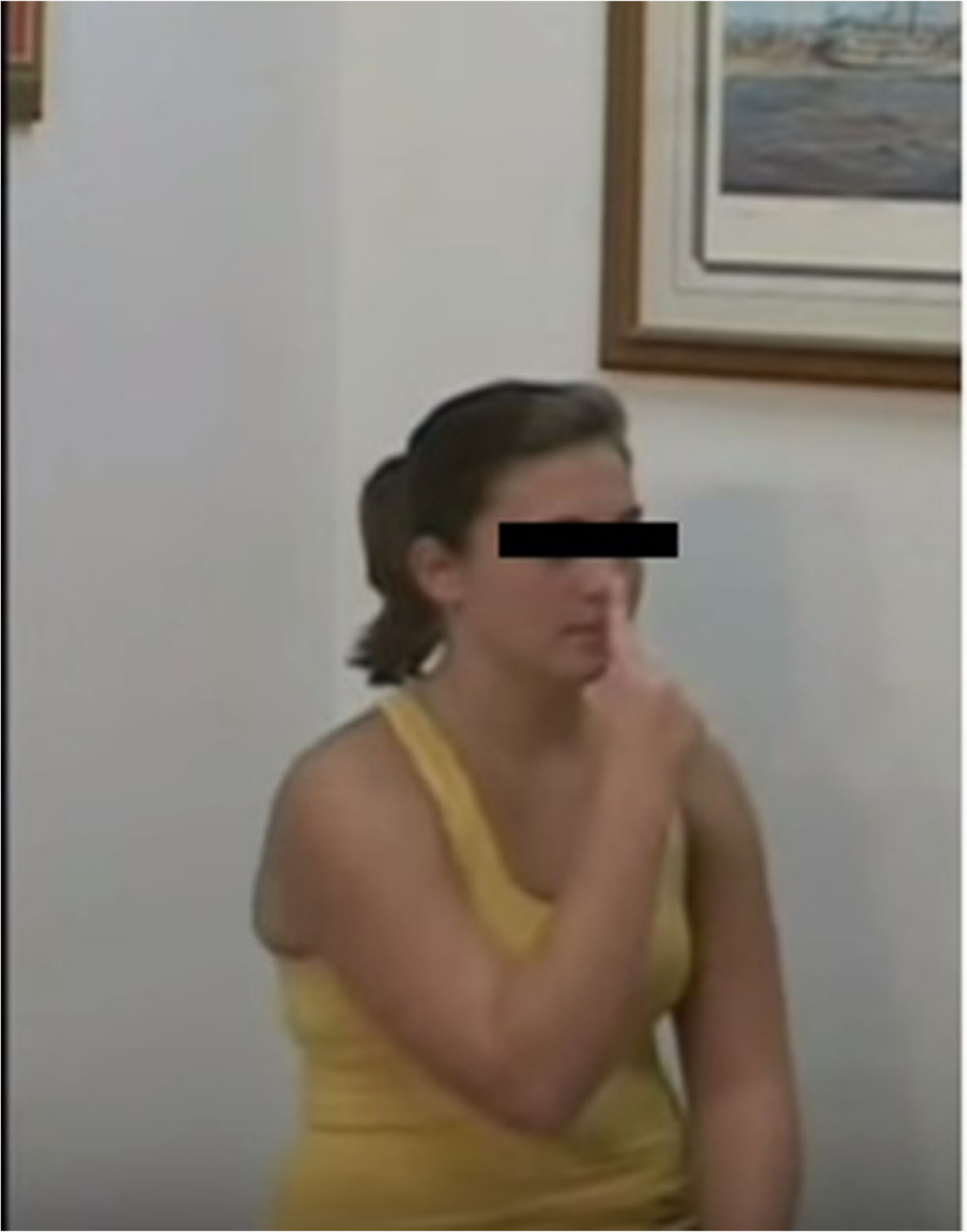
Finger to nose maneuver

**Fig. 2:**
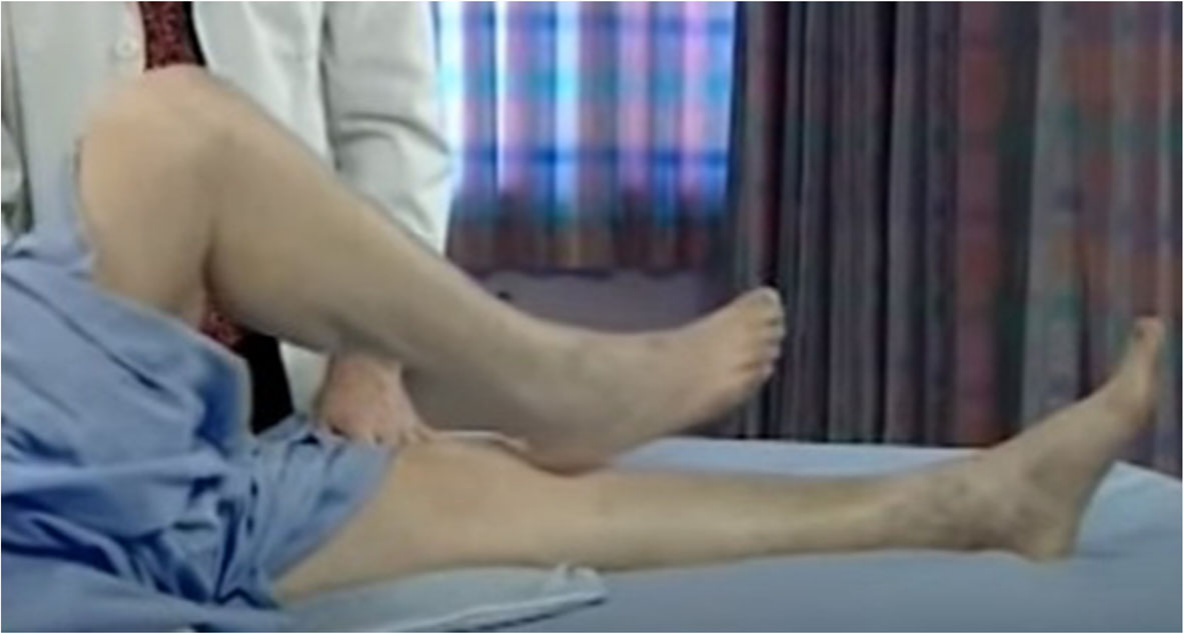
Heal to knee maneuver

Typical signs and specific maneuvers may be of great help in revealing childhood ataxia. This can be evaluated in various conditions: in a sitting position, in which the affected child manifests a loss of truncal control, and during walking, in which the patient exhibits a tandem gait or a veering toward the affected side. The recognition of ataxia is particularly difficult in early childhood. The most common cerebellar symptom is unsteadiness of gait. The child stands with feet widely separated and rapidly lose his balance. On attempting to walk, the child sways and stops, and may walk backward. In some cases, incoordination of the eye movements may be present. After the age of 3 years, the semiology of ataxia is similar to that of adulthood. The coordination may be explored through typical maneuvers such as **finger to finger or finger to nose**, and rapid alternating hand movements (Fig. 1). **Heal to knee** dysregulation (Fig. 2) and dysmetria observed through errors in fixing the correct distance (too long or too short) may be of diagnostic value. Slurred speech, scarring and poor expression are signs of cerebellar involvement. Sensory tactile, painful, thermal, and profound evaluation must be registered; any abnormalities should accordingly be carefully checked.

**Fig. 3:**
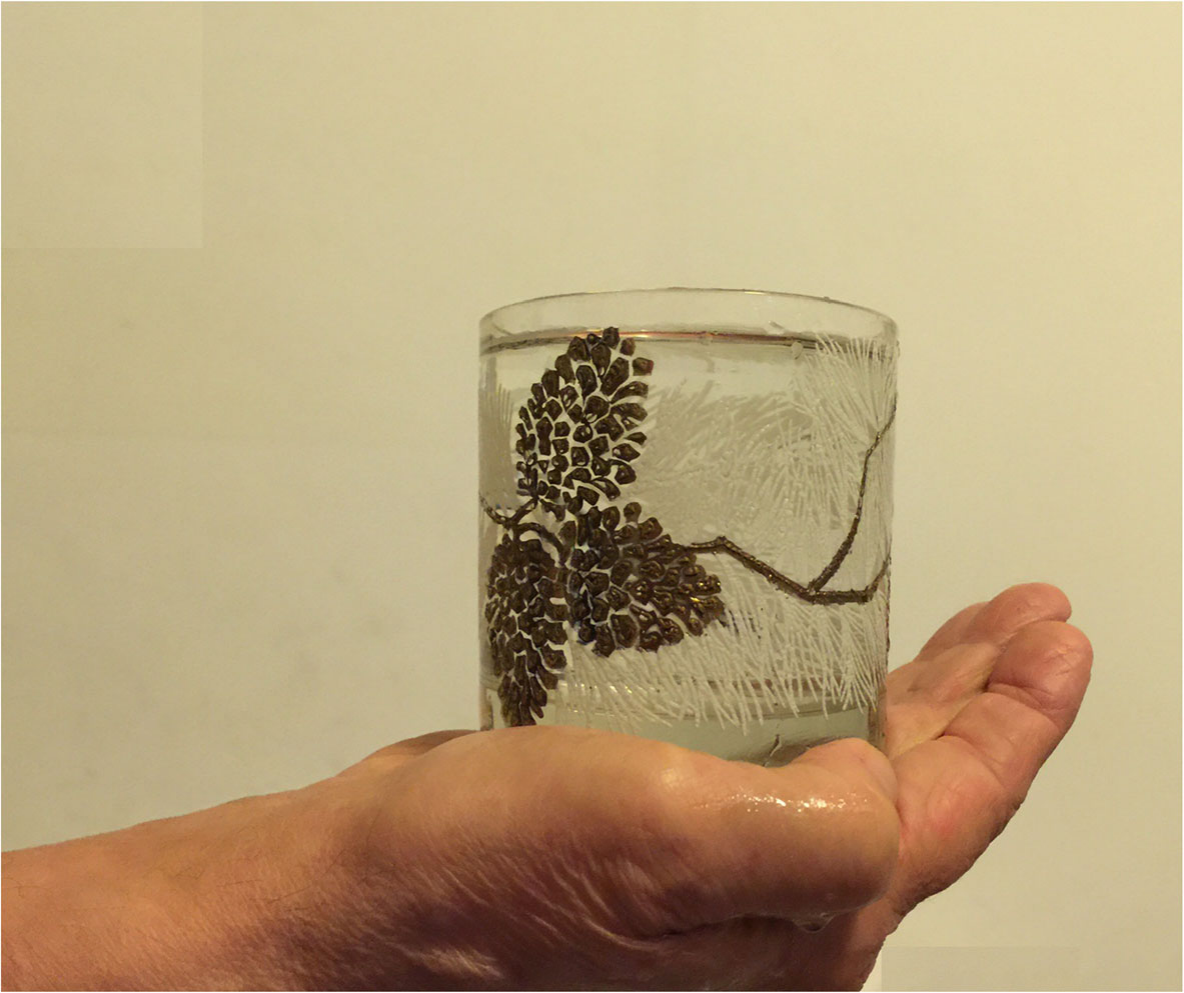
Specific maneuver consisting in holding a full glass of water

Two simple but useful maneuvers for revealing ataxia in children are the Romberg sign, expressed by the tendency to fall down with closed eyes in a holding position, and the test of **holding a full glass of water** with a steady hand without any of the water spilling (Fig. 3) [5, 6].

Many conditions may show ataxia as one of their primary clinical signs. In some case, ataxia may be expressed as a rapid and benign course, and it is sometimes manifested as a progressive and severe neurological involvement.

According to its etiology, ataxia may be displayed as a condition that is acquired, inherited or sporadic. The course of ataxia may be distinguished as being acute, intermittent and recurrent, chronic-non-progressive and chronic-progressive.

Temporal course distinction must be taken in a broad sense and with the awareness that the same disorder may have a different course, depending on the age of presentation, time of the diagnosis (precocious or late). This is particularly true for brain tumors that usually have a progressive course, but in very young children may present with a quite acute onset. At the same time, brainstem and cerebellar malformations are correctly included in the group of chronic non-progressive ataxias, but if the disorder is identified precociously, it could be included in the group of chronic progressive ones.

Ataxia in children as one of the main clinical signs of different disorders is reported in the following sections and discussed on the basis of its temporal course.

**Fig. 4:**
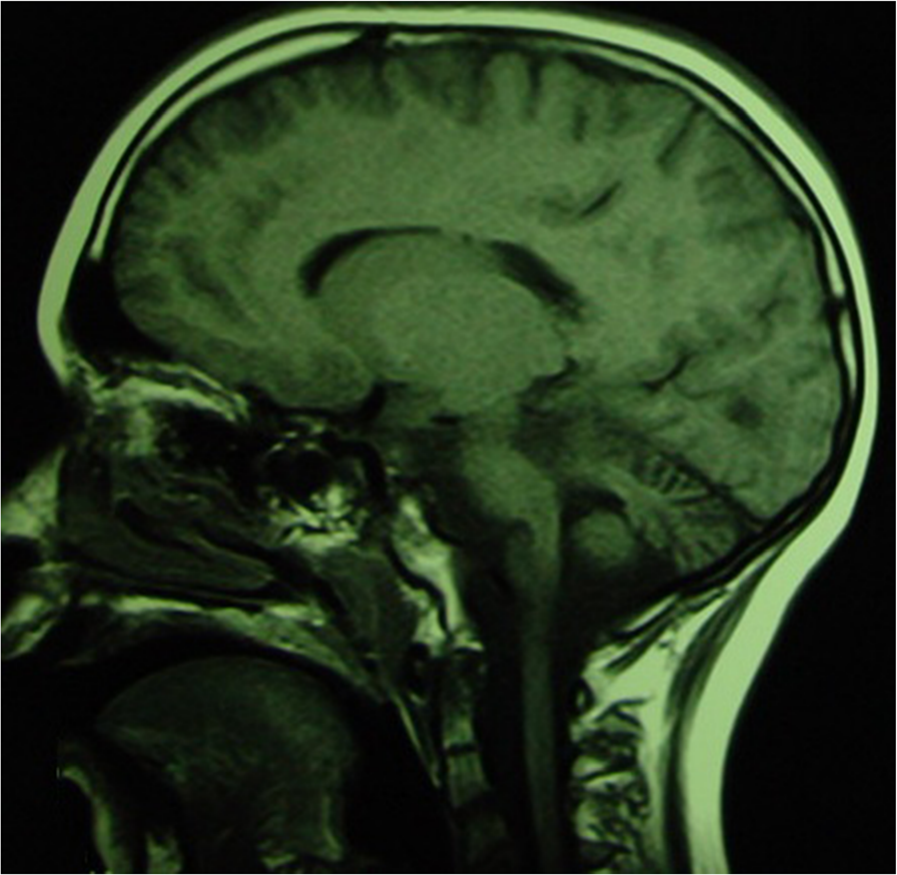
Sagittal T1-weighted MRI images of a 3-year-old aged patient presenting ponto-cerebellar dysplasia

**Fig. 5:**
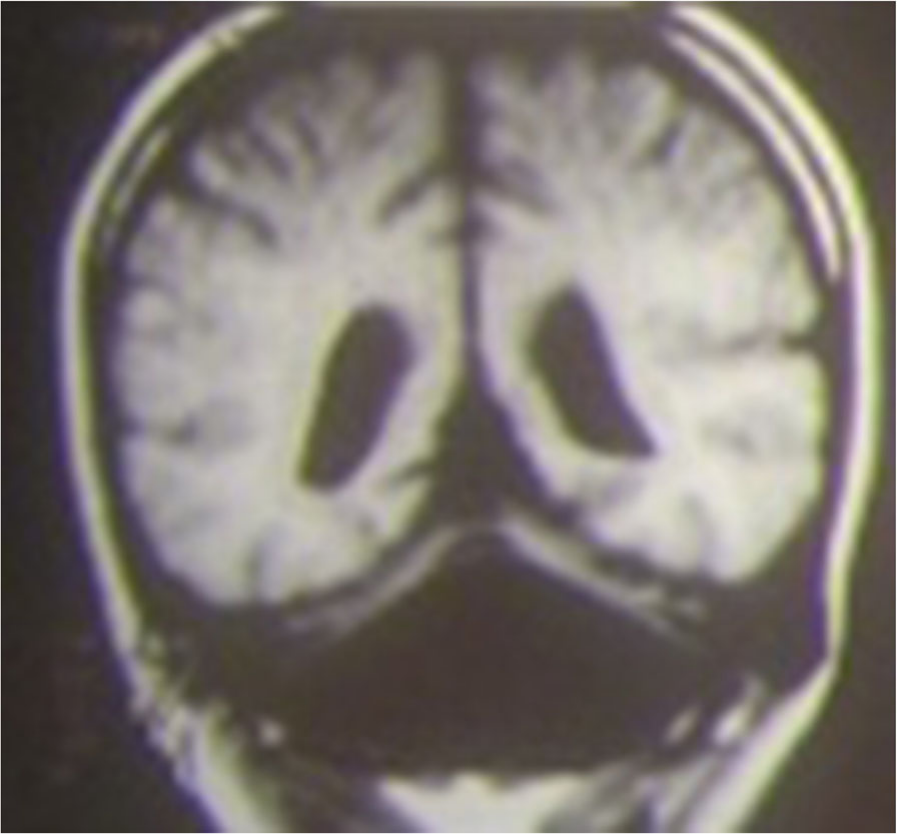
Coronal T1-weighted MRI image of an 8-year-old patient affected by cerebellar dysplasia. The cerebellum is markedly hypotrophic, and the lateral ventricles are wide
  Presently, more than 35 types of SCAs are recognized. The forms of SCAs are categorized according to the number assigned to the gene loci from *SCA1* to *SCA37*. **Dentato-rubral-pallidoluysian atrophy (DRPLA)** belongs to this group. Some of these forms are caused by the expansion of microsatellite repeats (SCA 1–3, 6–8, 10, 12, 17, 31, 36). Expansion of polyglutamine coding CAG repeats are related to the forms SCAs 1–3, 6, 7 and 17, and the expansion of non-coding repeats are related to SCAs 8, 10, 12, 31 and 36. Point mutations are linked to the other forms. Ataxia, with a variable age of onset, from congenital to adult, and a variable course ranging from non-progressive to slowly progressive, is related to a single type of SCA. Associations with ophthalmologic and auditory anomalies, hypotonia, cognitive delay and sensory anomalies may be a component of each of these forms [1, 37–41]. SCA type 3 (**Machado-Joseph disease**) is a well-known disorder. Affected patients exhibit both ataxia and pyramidal signs and peripheral amyotrophy, nystagmus, ophthalmoparesis and bulging eyes. Fasciculations involve the face, tongue and limbs. Dystonia and movement disorders are also associated [4, 42].
  X-linked SCAs include the **Fragile X syndrome** associated with tremor/ataxia syndrome with mutations in the *FMR1* gene. Mutations in mitochondrial DNA may manifest themselves with ataxia, myopathy, external ophthalmoplegia, endocrine deficiencies, short stature and retinal pigmentary degeneration [43, 44].
  ARCAs are distinctive disorders linked to cerebellar and spinal cord degeneration. The most well-known ARCAs include **Friedreich’s ataxia (FA)** and **ataxia teleangectasia**. Both of these disorders are inherited with autosomal recessive inheritance. FA involves the chromosome 9 GAA trinucleotide expansion. This disorder strongly affects the afferent/sensory circuits and the cerebellum. Its primary symptoms consist of ataxia, pes cavus, scoliosis and areflexia. Hypertrophic cardiomyopathy is a relevant sign present in affected children [45, 46]. Ataxia teleangectasia involves chromosome 11. Over 200 mutations involving the *ATM* gene have been reported. The primary symptoms consist of progressive truncal ataxia, oculo-cutaneous teleangectasias, polyneuropathy, hypotonia and oculomotor apraxia. Teleangectasia is more frequently localized on the conjunctivae but is also found in ear lobes and the popliteal fossa (Fig. 6). Low levels of circulating immunoglobulins (IgA, IgM, IgG and IgE), together with lymphocytopenia and increased alpha-fetoprotein, are reported. Recurring infections of the upper and lower respiratory tracts have been quoted [47, 48]. **Ataxia with oculomotor apraxia (AOAs)** is a group of recessive disorders associated to FA. Children with AOAs manifest ataxia, oculomotor apraxia, polyneuropathy and, in some cases, also dysarthria, choreoatetosis and dystonia. Cognitive delay may be present. In patients with AOA type 2 axonal neuropathy, cerebellar atrophy and increased levels of alpha-fetoprotein are found. Movement disorders are also present [49, 50]. Ataxias are signs reported in other AR disorders such as **SeSAME syndrome** (seizures, sensoneural deafness, ataxia, mental retardation and electrolyte unbalance) and in **SYNC1 ataxia** (spinocerebellar ataxia, spinocerebellar atrophy, and cerebellar ataxia and coenzyme Q10 deficiency (ARCA2) [51, 52].

1. **Acute ataxia**
  The most common causes of acute ataxia in children are excessive drug ingestion, drug intoxications and post-infectious cerebellitis. Antiepileptic drugs that may cause ataxia include benzodiazepines (diazepam, clobazam, nitrazepam), oxcarbamazepine, lamotrigine and phenytoin [7–9] and antineoplastic/immunosuppressive agents such as cyclosporine, tacrolimus, cytosine arabinoside and similar drugs [1]. Ataxia may also be related to intoxications to various elements such as alcohol, ethylene glycol, lead, mercury, thallium, lithium and toluene [10, 11].
  Low intake of the vitamins thiamine, cobalamin, vitamin E, zinc and folate in neglected children or in children affected by intestinal disorders may results in symptoms of ataxia [1–3].
  Varicella is one of the primary infectious agents that affects the cerebellum. The disorder may hit children of all ages, but it is most prevalent between the ages of 2 and 8 years. In this case, ataxia begins 2–6 days after the onset of rash, but it may also occur during the incubation period or after resolution of the rash [12]. Among the other involved infectious agents, cerebellar impairment may manifest itself after mumps or infectious mononucleosis. Syphilis and Whiplle’s disease, HIV, and Kawasaki disease may also be causes of ataxia [1–3]. Labyrinthitis is often associated to ataxia and is difficult to differentiate from acute cerebellar ataxia.
  The recent discoveries on immunomediated disorders have increased the number of the various affections that exhibit signs of ataxia. One of the most controversial is the association of ataxia with antigliadin antibodies, so-called “gluten ataxia.” Children presumed to be affected by this disorder present gait ataxia, nystagmus, peripheral neuropathy and brain involvement upon MRI as a consequence of a gluten trigger. A gluten-free diet has been reported to improve the clinical signs in the affected patients [13]. In a systematic review and metanalysis led by Lionetti et al. [14], the presence of cerebellar gluten-ataxia was documented in two large studies and reported in 2.7 and 5.4% of patients, respectively; no correlation was found between coeliac patients and ataxia in two other study-analyses. Patients with malignancies—mainly patients with Hodgkin’s lymphoma and other types of neoplasms—may present ataxia. Anti-Purkinje cells, anti-Yo (type 1), anti Hu, and anti Ri antibodies have been reported in affected patients [15]. Antibodies to glutamic acid decarboxylase associated with thyroid disorders and/or insulin-dependent diabetes mellitus may manifest ataxia as one of the presenting signs [16], but this disorder is rare among children. In the group of thyroid disorders, the autoimmune thyroiditis also called Hashimoto encephalopathy may manifest with ataxia. This severe condition presents a progressive course and behavioral changes such as aggressivity and psychotic crises, sensory dulling, headache, memory deficits, tremor, myoclonus, and, in the most severe cases, coma [17, 18]. Ataxia is one of the components of ophthalmoplegia and areflexia of the triad of Miller-Fisher syndrome, which belongs to the group of Guillain-Barrè syndrome and is linked to the presence of the antibody anti CQ1B. Diagnosis of this syndrome is obtained via an analysis of cerebral liquor, which displays a dissociation between high CSF protein levels and a low or absent leucocyte response [19].
2. **Intermittent and remittent ataxia**
  Basilar migraine, “episodic ataxias” (EAs) including benign paroxysmal ataxia may manifest with ataxia. **Basilar migraine** is part of the subgroup of headaches, and intermittent ataxia is one of the signs of this condition, together with impaired vision, dysarthria, dizziness and occasional loss of consciousness. Familiarity, recurrence of headache episodes, and normality of the neurologic examination are elements that often lead to a diagnosis of basilar migraine [20]. **EAs** represent a clinically heterogeneous groups of conditions with recurrent bouts of abrupt-onset of ataxia lasting minutes to hours. Clinical manifestations may also include vomiting, vertigo, headache, dysarthria, diplopia and dystonic attacks. Eight subtypes have been recently distinguished according to clinical and genetic characteristics and 5 genes are recognized to be linked to EAs more common being KCNA1 and CACNA1A genes mutations [21]. Some of these disorders, mainly the types 2, 3, and 5 EAs have shown a good response to treatment with acetazolamide [22].
  Some inborn errors of metabolism may present with intermittent ataxia. An example of this condition is **Hartnup disease**, an autosomal recessive (AR)-inherited disorder due to an abnormal renal and gastrointestinal transport of neutral amino acids. The primary sign of this disorder is cutaneous photosensitivity. Sun exposure in affected children causes cutaneous redness, and a pellagra-like rash that is severely pruritic. Some patients show an intermittent, unsteady, wide-based gait. Prognosis is usually benign [23–25]. **Maple syrup urine disease (MSUD)** is an AR disorder that involves branched-chain amino acids. The diagnosis is based on the classical sweet smell of maple syrup of the urines. Several phenotypes of MSUD are recognized: in the intermittent MSUD type during stress or an infectious episode, children may present vomiting and ataxia. When not treated, the disorder can lead to lethargy until coma [26, 27]. **Pyruvate dehydrogenase complex** deficiency is typically a severe disorder with different clinical aspects and biochemical presentations. In older children, the enzyme activity may be partially present and result in mild-to-moderate hyperlactic acidemia and ketoacidosis following stress, infectious episodes and high carbohydrate ingestion. This disorder is clinically associated with frequent episodes of ataxia, vomiting and breathing difficulties [28, 29].
  Mutations in genes coding for ion channels and transport proteins are associated with hereditary episodic ataxia and vertigo [30, 31].
3. **Chronic-non-progressive ataxias**
  This group includes the outcome of patients suffering from **stroke** and **hypoxic-ischemic encephalopathy** outcome. This condition is the most frequent cause of cerebral damage with an incidence rate of 1.5 individuals per 1000 newborns. The cerebral involvement is linked in about 90% of cases to perinatal asphyxia that acts through two linked pathogenetic mechanisms: hypoxia with reduced hematic O_2_ concentration and ischemia with lowered cerebral perfusion [32]. The affected children show several manifestations with cognitive delay, epileptic seizures, spasticity, impairment of voluntary movements and coordination and neuro-behavioral anomalies such as aggressivity and restlessness [3].
  Chronic ataxic manifestations that are typically not progressive may be found within two cerebral malformation syndromes: **Dandy-Walker syndrome** (DWS) and **Arnold-Chiari malformation** (ACM). The first syndrome is characterized by an enlargement of the fourth ventricle, the complete absence of cerebellar vermis and cystic formation near the internal base of the skull. In ACM, the affected children show a downwards displacement of the cerebellar tonsils through the foramen magnum with a presumed risk to complicate with a non-obstructive hydrocephalus.
  Multiple sclerosis in young children may initially manifest as intermittent ataxia [1, 3, 6].
  Ataxia is one of the signs indicative of cerebellar malformation in which the cerebellum can be totally or partially involved. The most well-known form is **Joubert’s syndrome**, an AR disorder due to a congenital ponto-cerebellar hypoplasia (PCH). Affected patients present neonatal hypotonia, ocular motor apraxia, breathing difficulties and multiple systemic involvement. More than 20 causative genes of Joubert’s syndrome have been recognized, most of which are related to proteins of the primary cilium, a subcellular organelle that carries out specific cellular functions. Diagnosis may be based on a typical sign of the molar tooth observed in the brain MRI of affected patients. The sign is related to the extension of the cerebellar superior peduncles in association with the depth interpeduncular furrow that resembles the shape of a molar tooth [33, 34].
  Lack of development and/or early neurodegeneration of cerebellum and brainstem are the main characteristics of PCH. These conditions encompasses a constellation of genetic conditions with involvement of several genes and distinctive clinic-radiological phenotypes [35].
  The pathologic anomalies of PCH consist of distinctive features including a reduced number of cellular folia and poor folial branching, degeneration and disappearance of pontine nuclei, fragmentation of dentate nuclei, variable degenerative changes and poor branching of the inferior olivar nuclei [36].
4. **Cerebellar progressive ataxias**.
  Inherited ataxias include a heterogenous group of clinically and genetically distinguished neurodegenerative disorders. The most well-known of the inherited ataxias include autosomal dominant cerebellar ataxia reported as **spino-cerebellar ataxias (SCAs)** and autosomal recessive cerebellar ataxias (ARCAs). X-linked inherited forms and ataxia associated with mithochondrial disorders are also included in this group (Figs. 4 and 5).

**Fig. 6:**
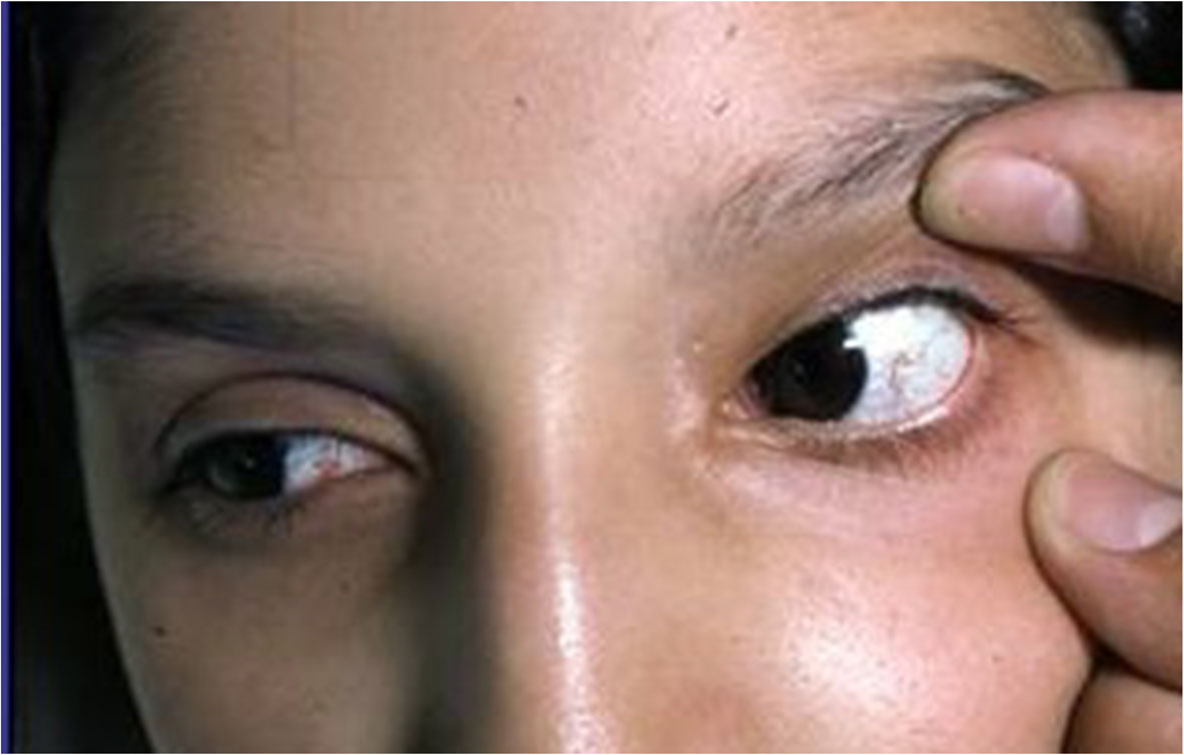
Typical teleangectasias localized on the conjunctivae of a 7 year-old patient
  Recently, cerebellar ataxia has been linked to mutations in the *PEX10* gene related to peroxisomal biogenesis disorders in a young patient with marked cerebellar atrophy [53].
  Autosomal recessive spastic ataxia of **Charlevoix Seguenay** is a complex disorder characterized by spasticity, ataxia, polyneuropathy and amyotrophy of distal muscles. Chromosome 13 is involved with over 70 mutations that have been reported in the *SAC* genes [54–56]. **Marinesco-Sjogren syndrome** exhibit cerebellar ataxia, myopathy with progressive muscular hypotonia, cognitive delay and congenital cataracts, often associated with skeletal deformities, short stature and hypogonadotropic hypogonadism [57, 58].
  Congenital errors of metabolism may show ataxia among the other progressive severe neurologic involvement. Examples of these disorders include some types of gangliosidoses, lipidoses and adrenoleukodystrophy. **Abetalipoproteinemia (Bassen-Kornzeig disease)** is an AR disorder linked to a mutation in microsomal triglyceride transfer protein (MTTP) genes. Steatorrhea and a failure to thrive are the initial signs later followed by ataxia, retinitis pigmentosa, peripheral neuritis, muscle weakness and cognitive delay. A blood smear is diagnostic with acanthocytosis together with low blood levels of cholesterol and triglycerides and the absence of beta-lipoproteins. The disorder is associated with malabsorption of lipids and lipid-soluble vitamins (i.e. A, D, E, and K) [59].
**Cerebral tumors**, particularly medulloblastoma, cerebellar astrocytoma, brain stem glioma and ependymomas, are among the most common causes of progressive ataxia in children [31]. The clinical onset of cerebral tumors is frequently preceded by episodes of headaches and vomiting followed rapidly by additional signs or symptoms. Warming signs beyond morning vomiting and headaches include specific neurologic symptoms such as seizures, vision difficulties and frontal and occipital pain. There is a progression of the symptoms both in frequency and intensity with exacerbation given a change of position or movement. Mood changes with irritability, aggressivity and nocturnal awakening are also relevant diagnostic signs. Ataxia associated with myoclonus and opsoclonus is a characteristic sign of medulloblastoma [60, 61]. In all the types of cerebral tumors, brain imaging represents the best way to obtain a correct diagnosis.

### Treatment

Some advances have been obtained in some of the disorders with ataxia. Treatment of EAs with acetazolamide and GLT1SD with ketogenic diet gave good results [21, 22]. However, a really few hereditary diseases associated with ataxia may be fully treatable. Symptomatic treatment and supportive management may alleviate the course of these severe disorders.

## Conclusions

**Fig. 7:**
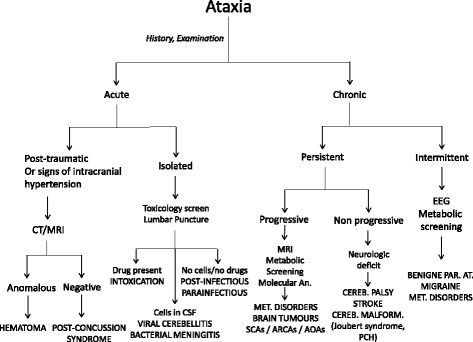
Clinical flow-chart of Ataxia

Recognizing ataxia in children may be challenging. It may be overlooked mainly in very young children and erroneously related to a delay of coordination. Physical examination and correct maneuvers are useful for highlighting its clinical sign. The causal events of ataxia are several and have different outcomes that may range from transient and benign to particularly severe and frightening. Pediatricians must be skilled at differentiating treatable disorders from progressive, degenerative and devastating ones, some of which are singularly difficult to diagnose. For most of these disorders, there is no curative care available; only supportive treatment is suitable. A flowchart of the primary causes of ataxia is listed in Fig. 7.
